# Supplementary material for: Trajectories of healthy ageing among older adults with multimorbidity: A growth mixture model using harmonised data from eight ATHLOS cohorts
Source: PLoS One. 2021 Apr 6;16(4):e0248844. doi: 10.1371/journal.pone.0248844 (PMC8023455; doi:10.1371/journal.pone.0248844)
Supplement: S1 Table — (DOCX) [file pone.0248844.s001.docx]

**Supplement Table S1: 41 items related to intrinsic capacity and functional ability used in the harmonisation process**


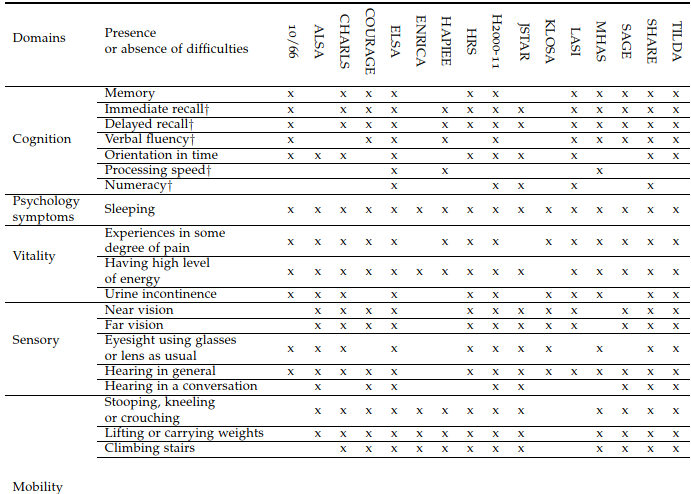


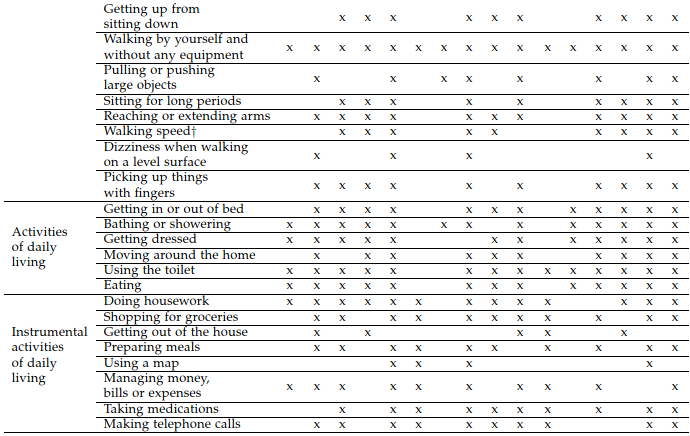


Source: ATHLOS technical report 2019. NB: items marked ꝉ were measured by performance tests (otherwise self-reported)
