# Supplementary material for: Trajectories of healthy ageing among older adults with multimorbidity: A growth mixture model using harmonised data from eight ATHLOS cohorts
Source: PLoS One. 2021 Apr 6;16(4):e0248844. doi: 10.1371/journal.pone.0248844 (PMC8023455; doi:10.1371/journal.pone.0248844)
Supplement: S2 Table — (DOCX) [file pone.0248844.s002.docx]

**Supplement Table S2: Baseline characteristics of analytic and excluded samples**

|  | **Analytic sample N = 130880** | **Excluded sample N = 73296** | **t/X^2^** | **Effect size** |
| --- | --- | --- | --- | --- |
| **Study, n (%)** |  |  |  |  |
| ALSA | 1851 (1.4) | 236 (0.3) | 27000** | 0.36 |
| ELSA | 14498 (11.1) | 3501 (4.8) |  |  |
| ENRICA | 2516 (1.9) | 3 (0.0) |  |  |
| HRS | 32988 (25.2) | 3821 (5.2) |  |  |
| JSTAR | 3695 (2.8) | 3571 (4.9) |  |  |
| KLOSA | 8928 (6.8) | 1326 (1.8) |  |  |
| MHAS | 12925 (9.9) | 7409 (10.1) |  |  |
| SHARE | 53479 (40.9) | 53429 (72.9) |  |  |
| **Age, mean (SD)** | 63.4 (10.2) | 62.8 (10.1) | 29.89** | 0.15 |
| **Sex, n (%)** |  |  |  |  |
| Female | 72497 (55.5) | 34720 (54.1) | 35.57** | -0.01 |
| Male | 58238 (44.5) | 29414 (45.9) |  |  |
| **Education, n (%)** |  |  |  |  |
| Less than primary/primary | 43641 (34.0) | 17805 (27.4) | 622.06** | 0.06 |
| Secondary | 63052 (49.1) | 34065 (52.4) |  |  |
| Tertiary | 21780 (16.9) | 13146 (20.2) |  |  |
| **Wealth, n (%)** |  |  |  |  |
| Quintile 1 (lowest) | 24873 (19.9) | 11206 (19.3) | 17.20* | 0.01 |
| Quintile 2 | 24528 (19.7) | 10899 (18.7) |  |  |
| Quintile 3 | 24933 (20.0) | 11428 (19.6) |  |  |
| Quintile 4 | 24704 (19.8) | 12007 (20.6) |  |  |
| Quintile 5 (highest) | 25683 (20.6) | 12637 (21.7) |  |  |
| **Smoking, n (%)** |  |  |  |  |
| Ever smoked | 64321 (49.6) | 27481 (45.2) | 107.64** | 0.03 |
| Never smoked | 65307 (50.4) | 33354 (54.8) |  |  |
| **Drinking, n (%)** |  |  |  |  |
| Often | 31902 (27.1) | 16163 (27.7) | 89.15** | 0.02 |
| Rare | 44277 (37.6) | 22126 (37.9) |  |  |
| Never | 41635 (35.3) | 20148 (34.5) |  |  |
| **Physical activity, n (%)** |  |  |  |  |
| Sedentary/low | 42225 (38.5) | 16347 (31.2) | 291.88** | 0.05 |
| Moderate | 40822 (37.2) | 20335 (38.8) |  |  |
| High | 36744 (24.3) | 15723 (30.0) |  |  |
| **Multimorbidity, n (%)** |  |  |  |  |
| Presence | 32818 (25.1) | 14527 (21.9) | 57.29** | -0.02 |
| Absence | 98042 (74.9) | 51870 (78.1) |  |  |

Effect size for age corresponded to Cohen’s d while the remaining effect size estimates corresponded to Cramer’s V. All effect size estimates were computed with non-missing data only. N=number, SD=standard deviation, t=student’s t-test, X^2^=chi-square test, *: p-value <0.05, **: p-value <0.001
