# Supplementary material for: Trajectories of healthy ageing among older adults with multimorbidity: A growth mixture model using harmonised data from eight ATHLOS cohorts
Source: PLoS One. 2021 Apr 6;16(4):e0248844. doi: 10.1371/journal.pone.0248844 (PMC8023455; doi:10.1371/journal.pone.0248844)
Supplement: S3 Table — Unadjusted GMM model using data from the first three waves of the ATHLOS harmonised dataset. (DOCX) [file pone.0248844.s003.docx]

**Supplement Table S3:**

**Model fit information – linear growth mixture model using the first three waves of the ATHLOS harmonised dataset**

| **Number of classes** | **2 classes** | **3 classes** | **4 classes** | **5 classes** |
| --- | --- | --- | --- | --- |
| Sample size | 89740 | 89740 | 89740 | 89740 |
| Number of parameters | 11 | 14 | 17 | 20 |
| AIC | 1416646 | 1415298 | 1414306 | 1413374 |
| BIC | 1416749 | 1415430 | 1414466 | 1413562 |
| SABIC | 1416714 | 1415386 | 1414412 | 1413499 |
| LMR LR p-value | <0.001 | <0.001 | <0.001 | <0.001 |
| aLMR LR p-value | <0.001 | <0.001 | <0.001 | <0.001 |
| BLRT p-value | <0.001 | <0.001 | <0.001 | <0.001 |
| Entropy | 0.62 | 0.69 | 0.65 | 0.67 |
| Class size (%) |  |  |  |  |
| Class 1 | 79% | 77% | 34% | 38% |
| Class 2 | 21% | 21% | 7% | 16% |
| Class 3 |  | 2% | 57% | 2% |
| Class 4 |  |  | 2% | 1% |
| Class 5 |  |  |  | 42% |

AIC = Akaike information criteria, BIC = Bayesian information criteria, aBIC = adjusted Bayesian information criteria, LMR LR = Vuong-Lo-Mendell-Rubin likelihood ratio test, aLMR LR = adjusted Lo-Mendell-Rubin likelihood ratio test, BLRT = bootstrapped likelihood ratio test.

**Unadjusted GMM model using data from the first three waves of the ATHLOS harmonised dataset**

|  | **High stable** | **Low stable** | **Rapid decline** |
| --- | --- | --- | --- |
| N (%) | 69419 (77.4) | 18906 (21.1) | 1415 (1.6) |
| Mean intercept (SE) | 54.35 (0.06) | 41.02 (0.10) | 55.79 (0.23) |
| Mean slope (SE) | -0.33 (0.04) | -0.71 (0.05) | -10.22 (0.31) |
| Variance intercept (SE) | 22.79 (0.53) | | |
| Variance linear term (SE) | 0.94 (0.33) | | |
| Covariance intercept linear term (SE) | 0.90 (0.33) | | |

N = number, SE = standard error
