# Supplementary material for: Trajectories of healthy ageing among older adults with multimorbidity: A growth mixture model using harmonised data from eight ATHLOS cohorts
Source: PLoS One. 2021 Apr 6;16(4):e0248844. doi: 10.1371/journal.pone.0248844 (PMC8023455; doi:10.1371/journal.pone.0248844)
Supplement: S4 Table — (DOCX) [file pone.0248844.s004.docx]

**Supplement Table S4: ELSA sample characteristics Waves 2 - 7**

|  | **Wave 2 N = 9171** | **Wave 3 N = 7880** | **Wave 4 N = 7347** | **Wave 5 N = 7381** | **Wave 6 N = 7153** | **Wave 7 N = 6423** |
| --- | --- | --- | --- | --- | --- | --- |
| **Age, mean (SD)** | 66.4 (10.3) | 66.5 (10.3) | 66.7 (10.3) | 66.7 (10.4) | 66.8 (10.5) | 66.5 (10.5) |
| **Sex, n (%)** |  |  |  |  |  |  |
| Male | 4084 (44.5) | 3505 (44.5) | 3271 (44.5) | 3306 (44.8) | 3211 (44.9) | 2885 (44.9) |
| Female | 5087 (55.5) | 4375 (55.5) | 4076 (55.5) | 4075 (55.2) | 3942 (55.1) | 3538 (55.1) |
| **Disease group, n (%)** |  |  |  |  |  |  |
| Cardiorespiratory/arthritis/cataracts | 1826 (19.9) | 1567 (19.9) | 1487 (20.2) | 1512 (20.5) | 1481 (20.7) | 1297 (20.2) |
| Metabolic | 844 (9.2) | 736 (9.3) | 678 (9.2) | 678 (9.2) | 650 (9.1) | 579 (9.0) |
| Relatively healthy | 6501 (70.9) | 5577 (70.8) | 5182 (70.5) | 5191 (70.3) | 5022 (70.2) | 4547 (70.8) |
| **Healthy ageing, mean (SD)** | 49.1 (9.4) | 48.8 (9.3) | 48.5 (9.2) | 48.4 (9.5) | 48.0 (9.5) | 48.2 (9.6) |
| **Education, n (%)** |  |  |  |  |  |  |
| Higher | 2236 (24.4) | 2016 (25.6) | 1893 (25.8) | 1923 (26.1) | 1868 (26.1) | 1715 (26.7) |
| Intermediate | 3415 (37.2) | 2953 (37.5) | 2748 (37.4) | 2747 (37.2) | 2654 (37.1) | 2416 (37.6) |
| No degree | 3506 (38.2) | 2903 (36.8) | 2699 (36.7) | 2705 (36.6) | 2624 (36.7) | 2285 (35.6) |
| **Net financial health, n (%)** |  |  |  |  |  |  |
| quintile 1 (lowest) | 1708 (18.6) | 1412 (17.9) | 1315 (17.9) | 1313 (17.8) | 1285 (18.0) | 1131 (17.6) |
| quintile 2 | 1600 (17.4) | 1350 (17.1) | 1244 (16.9) | 1227 (16.6) | 1209 (16.9) | 1052 (16.4) |
| quintile 3 | 1726 (18.8) | 1512 (19.2) | 1399 (19.0) | 1430 (19.4) | 1373 (19.2) | 1215 (18.9) |
| quintile 4 | 1787 (19.5) | 1568 (19.9) | 1469 (20.0) | 1490 (20.2) | 1429 (20.0) | 1322 (20.6) |
| quintile 5 (highest) | 1840 (20.1) | 1646 (20.9) | 1556 (21.2) | 1564 (21.2) | 1518 (21.2) | 1393 (21.7) |
| **Smoking status, n (%)** |  |  |  |  |  |  |
| Smoker/ex-smoker | 5731 (62.5) | 4922 (62.5) | 4591 (62.5) | 4633 (62.8) | 4489 (62.8) | 4023 (62.6) |
| Non-smoker | 3276 (35.7) | 2851 (36.2) | 2648 (36.0) | 2642 (35.8) | 2560 (35.8) | 2305 (35.9) |
| **Drinking, n (%)** |  |  |  |  |  |  |
| Regular | 2816 (30.7) | 2475 (31.4) | 2292 (31.2) | 2303 (31.2) | 2247 (31.4) | 2032 (31.6) |
| Occasional | 3528 (38.5) | 3061 (38.8) | 2849 (38.8) | 2870 (38.9) | 2743 (38.3) | 2503 (39.0) |
| Never | 1577 (17.2) | 1362 (17.3) | 1274 (17.3) | 1270 (17.2) | 1235 (17.3) | 1065 (16.6) |
| **Physical activity, n (%)** |  |  |  |  |  |  |
| Sedentary/low | 2807 (30.6) | 2388 (30.3) | 2221 (30.2) | 2275 (30.8) | 2193 (30.7) | 1955 (30.4) |
| Moderate | 4552 (49.6) | 3914 (49.7) | 3653 (49.7) | 3631 (49.2) | 3522 (49.2) | 3136 (48.8) |
| High | 1682 (18.3) | 1475 (18.7) | 1372 (18.7) | 1379 (18.7) | 1340 (18.7) | 1244 (19.4) |

N = number, SD = standard deviation
