# Supplementary material for: Trajectories of healthy ageing among older adults with multimorbidity: A growth mixture model using harmonised data from eight ATHLOS cohorts
Source: PLoS One. 2021 Apr 6;16(4):e0248844. doi: 10.1371/journal.pone.0248844 (PMC8023455; doi:10.1371/journal.pone.0248844)
Supplement: S5 Table — (DOCX) [file pone.0248844.s005.docx]

**Supplement table S5: Model fit information – linear growth mixture model for ELSA dataset**

| **Number of classes** | **2 classes** | **3 classes** | **4 classes** | **5 classes** |
| --- | --- | --- | --- | --- |
| Sample size | 9171 | 9171 | 9171 | 9171 |
| Number of parameters | 14 | 17 | 20 | 23 |
| AIC | 252706 | 252287 | 252185 | 252141 |
| BIC | 252806 | 252408 | 252327 | 252305 |
| SABIC | 252761 | 252354 | 252264 | 252232 |
| LMR LR p-value | <0.0001 | <0.0001 | 0.2639 | 0.3929 |
| aLMR LR p-value | <0.0001 | <0.0001 | 0.2722 | 0.3979 |
| BLRT p-value | <0.0001 | <0.0001 | <0.0001 | <0.0001 |
| Entropy | 0.66 | 0.73 | 0.71 | 0.70 |
| Class size |  |  |  |  |
| Class 1 | 62% | 3% | 36% | 0% |
| Class 2 | 38% | 36% | 4% | 7% |
| Class 3 |  | 61% | 1% | 2% |
| Class 4 |  |  | 58% | 36% |
| Class 5 |  |  |  | 55% |

AIC = Akaike information criteria, BIC = Bayesian information criteria, aBIC = adjusted Bayesian information criteria, LMR LR = Vuong-Lo-Mendell-Rubin likelihood ratio test, aLMR LR = adjusted Lo-Mendell-Rubin likelihood ratio test, BLRT = bootstrapped likelihood ratio test.
